# γ-Nanofluid Thermal Transport between Parallel Plates Suspended by Micro-Cantilever Sensor by Incorporating the Effective Prandtl Model: Applications to Biological and Medical Sciences

**DOI:** 10.3390/molecules25081777

**Published:** 2020-04-13

**Authors:** Umar Khan, Adnan, Naveed Ahmed, Syed Tauseef Mohyud-Din, Yu-Ming Chu, Ilyas Khan, Kottakkaran Sooppy Nisar

**Affiliations:** 1 Department of Mathematics and Statistics, Hazara University, Mansehra 21120, Pakistan; umar_jadoon4@yahoo.com; 2 Department of Mathematics, Mohi-ud-Din Islamic University, Nerian Sharif AJ&K 12080, Pakistan; adnan_abbasi89@yahoo.com; 3 Department of Mathematics, Faculty of Sciences, HITEC University, Taxila Cantt 47070, Pakistan; nidojan@gmail.com; 4 Department of Mathematics, University of Multan, Multan 60000, Pakistan; syedtauseefs@hotmail.com; 5 Department of Mathematics, Huzhou University, Huzhou 313000, China; chuyuming@zjhu.edu.cn or; 6 Faculty of Mathematics and Statistics, Ton Duc Thang University, Ho Chi Minh City 72915, Vietnam; 7 Department of Mathematics, College of Arts and Sciences, Prince Sattam bin Abdulaziz University, Wadi Aldawaser 11991, Saudi Arabia; n.sooppy@psau.edu.sa

**Keywords:** γAl_2_O_3_ nanoparticles, micro-cantilever sensor, numerical thermal transport, nanofluids effective models, thermophysical characteristics

## Abstract

The flow of nanofluid between infinite parallel plates suspended by micro-cantilever sensors is significant. The analysis of such flows is a rich research area due to the variety of applications it has in chemical, biological and medical sciences. Micro-cantilever sensors play a significant role in accurately sensing different diseases, and they can be used to detect many hazardous and bio-warfare agents. Therefore, flow water and ethylene glycol (EG) composed by γ-nanoparticles is used. Firstly, the governing nanofluid model is transformed into two self-similar nanofluid models on the basis of their effective models. Then, a numerical method is adopted for solution purposes, and both the nanofluid models are solved. To enhance the heat transfer characteristics of the models, the effective Prandtl model is ingrained in the energy equation. The velocity F’(η) decreases with respect to the suction of the fluid, because more fluid particles drags on the surface for suction, leading to an abrupt decrement in F’(η). The velocity F’(η) increases for injection of the fluid from the upper end, and therefore the momentum boundary layer region is prolonged. A high volume fraction factor is responsible for the denser characteristics of the nanofluids, due to which the fluids become more viscous, and the velocity F’(η) drops abruptly, with the magnetic parameters favoring velocity F’(η). An increase in temperature β(η) of Al_2_O_3_-H_2_O and γAl_2_O_3_-C_2_H_6_O_2_ nanofluids was reported at higher fraction factors with permeable parameter effects. Finally, a comparative analysis is presented by restricting the flow parameters, which shows the reliability of the study.

## 1. Introduction

The study of heat transfer in conventional as well as nanofluids between parallel plates suspended by micro-cantilever sensors is a research area with great potential, due to its wide range of applications in biological and medical sciences. It is a fact that conventional liquids are fluids with less heat transfer. For many industrial productions and engineering purposes, huge amounts of heat transfer are required, and conventional fluids are not capable of producing such amounts of heat.

The flow over a sensor surface by incorporating different physical phenomena in the governing flow equations cannot be neglected. In 2004, Khaled and Vafai [1] presented the characteristics of a hydromagnetically squeezed flow between the sensor surfaces. They revealed the behavior of velocity, temperature and local heat transfer rate by altering different emerging dimensionless physical quantities. In 2017, Salahuddin et al. [2] revealed the flow properties of Carreau–Yasuda fluid over the sensor surface when imposing a magnetic field. Kandasamy et al. [3] reported the flow of nanofluids in the presence of an imposed magnetic field. They revealed the impacts of the porosity on the governing model and explored the effects on the velocity and temperature profiles. They analyzed different nanofluids on the basis of various host liquids and different shapes of nanoparticles like laminar, sphere, and cylinder. 

In 2017, Kumar et al. [4] introduced the flow of tangent hyperbolic type fluid over the sensor surface. They highlighted the effects of squeezed and unsteady parameters on the properties of the flow and also emerged the phenomena of variable thermal conductivity in the governing flow model. Haq et al. [5] revealed the heat transfer behavior, velocity and skin friction coefficient in nanofluid squeezed between parallel plates suspended by micro-cantilever sensors. Recently, Atif et al. [6] studied the flow of Carreau fluid over the sensor surface. They explored the influences of thermal radiations and variable thermal conductance on the flow regimes. They tackled the respective nonlinear flow model by means of numerical technique. Moreover, they encountered the influences exerted by squeezed flow index, Weissenberg parameter, Prandtl number, power law index, and magnetic number on the velocity and temperature profiles. 

The flow squeezed between the plates is a research direction with a great deal of potential, and researchers and engineers have concentrated on analyzing such flows in consideration of various flow conditions. In 2018, Sobamowo and Akinshilo [7] revealed the flow of nanofluids squeezed between parallel plates placed in a Cartesian coordinate frame. From this emerged the phenomenon of Lorentz forces in the momentum equation, revealing fascinating results for the velocity and temperature fields. For mathematical analysis of the model, they adopted the regular perturbation technique. In 2017, Khan et al. [8] reported three-dimensional squeezed flow of water and ethylene glycol (EG) suspended by γAl_2_O_3_ nanoparticles. They adopted a numerical scheme for mathematical analysis and tackled the nanofluid models separately. They reported the dominant behavior of temperature for γAl_2_O_3_-C_2_H_6_O_2_ nanofluid. Furthermore, graphical comparison provided for the sake of the validity of the employed mathematical technique. 

In 2008, Siddiqui et al. [9] reported the squeezed flow bounded by two parallel plates. Recently, Ahmed et al. [10] reported the flow of hybrid nanofluid squeezed between two Riga plates. They used numerical technique for mathematical treatment of the hybrid model, and ensured the validity of the scheme by comparing the results with existing once in the literature. Moreover, the influences of various physical phenomenon ingrained in the hybrid model are revealed for the velocity, temperature and local heat transfer rate over the domain of interest. Hosseinzadeh et al. [11] examined the squeezed flow of magnetized nanofluid between Riga plates. They observed that the temperature of the flowing fluid was enhanced by increasing the Brownian motion parameter, and that the concentration field drops. Moreover, higher values of the thermophoresis parameter reduced the concentration field. In 2015, Mohyud-Din et al. [12] revealed a rotating nanofluid flow model between parallel plates. For nondimensionalization of the model, they implemented suitable similarity variables and partial differentiation and obtained a nonlinear nanofluid flow model. Homotopy Analysis and RK methods were adopted for the solutions and graphical results for the flow regimes and other physical quantities. 

From a review of the literature, it is revealed that the flow γAl_2_O_3_-H_2_O and γAl_2_O_3_-C_2_H_6_O_2_ nanofluids between parallel plates suspended by micro-cantilever sensors have not yet been examined. Such flows are very significant from biological, chemical and medical points of view. Therefore, two different sorts of governing nanofluid models were taken between the plates. Similarity variables were used for the process of nondimensionalization, and two distinct nanofluid models were obtained, depending on the host fluids water and ethylene glycol (EG). After that, the models were treated numerically and the results were explored with respect to their flow regimes, the coefficient of skin friction, the thermophysical characteristics of the nanofluid effective models, and the local heat transfer rate. In the last section, core output of the analysis by varying different physical parameters are given.

## 2. Materials and Methods

### 2.1. Statement and Geometry of the Model

**Figure 1:**
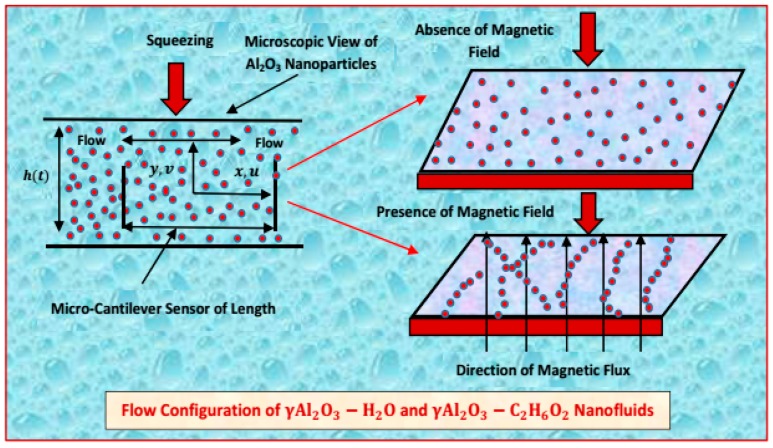
Flow of water and ethylene glycol (EG) suspended by ${\gamma \mathrm{Al}}_{2}O_{3}$ nanoparticles.

The flow of water and ethylene glycol (EG) composed of γAl_2_O_3_ nanoparticles is taken under consideration. It is assumed that the host is in thermal equilibrium with nanoparticles. The plates are placed inside a squeezing channel and separated by height h(t). A micro-cantilever sensor of length is suspended between the plates. Furthermore, the plate placed at lower end is fixed, while the plate at the upper end is squeezed. The flow of water and ethylene glycol (EG) composed of γAl_2_O_3_ nanoparticles is presented in Figure 1.

### 2.2. Governing Colloidal Model

The partial differential equations governing the flow of water and ethylene glycol (EG) dispersed by γAl_2_O_3_ nanoparticles are as presented in [1,3,5]:

(1) $$\frac{\partial \hat{u}}{\partial x}+\frac{\partial \hat{v}}{\partial y}=0,\;$$

(2) $$\frac{\partial \hat{u}}{\partial t}+\hat{u}\frac{\partial \hat{u}}{\partial x}+\hat{v}\frac{\partial \hat{u}}{\partial y}=\frac{1}{\rho_{nf}}\left(-\frac{\partial p}{\partial x}+\mu_{nf}\left(\frac{\partial^{2}\hat{u}}{\partial y^{2}}\right)-\sigma_{m}^{*}B_{m}^{2}\hat{u}\right),\;$$

(3) $$\frac{\partial \hat{U}}{\partial t}+\hat{U}\frac{\partial \hat{U}}{\partial x}=\frac{1}{\rho_{nf}}\left(-\frac{\partial p}{\partial x}-\sigma_{m}^{*}B_{m}^{2}\hat{U}\right),\;$$

(4) $$\frac{\partial \hat{T}}{\partial t}+\hat{u}\frac{\partial \hat{T}}{\partial x}+\hat{v}\frac{\partial \hat{T}}{\partial y}=\frac{{\hat{k}}_{nf}}{{\left(\hat{\rho }c_{p}\right)}_{nf}}\left(\frac{\partial^{2}\hat{T}}{\partial y^{2}}\right),\;$$

In the governing Equations (1)–(4), the velocities in the direction of x and y are represented by $\hat{u}$ and $\hat{v}$, respectively. The pressure is p, the magnetic field is $B_{m}^{2}$, the temperature is T, the specific heat capacitance is ${\left(\hat{\rho }c_{p}\right)}_{nf}$, the effective thermal conductivity is ${\hat{k}}_{nf}$, the effective density is $\rho_{nf}$, and $\mu_{nf}$ shows the dynamic viscosity. Furthermore, Equations (2) and (3) are the $\hat{u}$ component of the momentum equation and the component of the momentum equation for free stream, respectively. The conditions at the boundaries and away from them are given in the following way [5]:

(5) $$\begin{array}{c} \mathrm{The}\;\mathrm{conditions}\;\mathrm{for}\;\mathrm{the}\;\mathrm{velocity}\;\mathrm{at}\;\mathrm{the}\;\mathrm{surface}\;\mathrm{and}\;\mathrm{away}\;\mathrm{from}\;\mathrm{it}\\ \hat{u}\left(x,y,t\right){↓}_{y=0}=0,\\ \hat{v}\left(x,y,t\right){↓}_{y=0}=v_{0}\left(t\right),\\ \hat{u}\left(x,y,t\right){↓}_{y=0}=\hat{U}\left(x,t\right),\;t>0,\\ \mathrm{The}\;\mathrm{condition}\;\mathrm{for}\;\mathrm{temperature}\;\mathrm{at}\;\mathrm{the}\;\mathrm{surface}\\ \hat{T}\left(x,y,t\right){↓}_{y=0}=q\left(x\right),\;t>0,\\ \mathrm{The}\;\mathrm{condition}\;\mathrm{for}\;\mathrm{temperature}\;\mathrm{away}\;\mathrm{from}\;\mathrm{the}\;\mathrm{surface}\\ \hat{T}\left(x,y,t\right){↓}_{y=0}={\hat{T}}_{\infty } \end{array}\}$$

Here, ambient temperature is ${\hat{T}}_{\infty }$, heat flux is q(x), main stream velocity is $\hat{U}\left(x,t\right)$. For the problem under consideration, the applicable invertible transformations are given as:

(6) $$\begin{array}{c} \hat{U}=ax\\ \eta =\sqrt{\frac{ay^{2}}{\nu_{f}}}\\ a=\frac{1}{\left(s+bt\right)}\\ F\left(\eta \right)=\frac{\psi }{x\sqrt{a\nu_{f}}}\\ u=axF^{\prime }\left(\eta \right)\\ v=-\sqrt{a\nu_{f}}F\left(\eta \right)\\ \beta \left(\eta \right)=\frac{\hat{T}-{\hat{T}}_{\infty }}{\frac{q_{0}x}{k_{f}}\sqrt{\frac{\nu_{f}}{a}}}\\ q\left(x\right)=q_{0}\left(x\right)\\ v_{0}\left(t\right)=\nu_{f}\sqrt{a}\\ B_{m}\left(t\right)=B_{m0}\sqrt{a} \end{array}\}$$

The effective dynamic viscosity, thermal conductivity, heat capacitance, density, effective Prandtl number model, and the thermophysical characteristics of the nanofluids are revealed in [13]. By implementing the nanofluid models incorporated in [13], feasible similarity transformations and suitable partial derivatives in Equations (1)–(5), the following two nanofluid models are obtained on the basis of the host liquids water and ethylene glycol.

### 2.3. γAl_2_O_3_-H_2_O Model

(7) $$\frac{\left(123\phi^{2}+7.3\phi +1\right)}{\left(1-\phi +\frac{\phi {\hat{\rho }}_{s}}{{\hat{\rho }}_{f}}\right)}F^{\prime \prime \prime }+\left(F+\frac{\eta b}{2}\right)F^{\prime \prime }-F^{\prime 2}+b\left(F^{\prime }-1\right)+\frac{M}{\left(1-\phi +\frac{\phi {\hat{\rho }}_{s}}{{\hat{\rho }}_{f}}\right)}\left(1-F^{\prime }\right)+1=0,\;$$

(8) $$\beta^{\prime \prime }+\frac{\mathrm{Pr}\left(\left(1-\phi +\frac{\phi {\hat{\rho }}_{s}}{{\hat{\rho }}_{f}}\right)\left(82.1\phi^{2}+3.9\phi +1\right)\right)}{\left(123\phi^{2}+7.3\phi +1\right)}\left(\left(F+\frac{\eta b}{2}\right)\beta^{\prime }-\left(F^{\prime }+\frac{b}{2}\right)\beta \right)=0.\;$$

### 2.4. γAl_2_O_3_-C_2_H_6_O_2_ Model

(9) $$\frac{\left(306\phi^{2}-0.19\phi +1\right)}{\left(1-\phi +\frac{\phi {\hat{\rho }}_{s}}{{\hat{\rho }}_{f}}\right)}F^{\prime \prime \prime }+\left(F+\frac{\eta b}{2}\right)F^{\prime \prime }-F^{\prime 2}+b\left(F^{\prime }-1\right)+\frac{M}{\left(1-\phi +\frac{\phi {\hat{\rho }}_{s}}{{\hat{\rho }}_{f}}\right)}\left(1-F^{\prime }\right)+1=0,\;$$

(10) $$\beta^{\prime \prime }+\frac{\mathrm{Pr}\left(\left(1-\phi +\frac{\phi {\hat{\rho }}_{s}}{{\hat{\rho }}_{f}}\right)\left(254.3\phi^{2}-3.0\phi +1\right)\right)}{\left(306\phi^{2}-0.19\phi +1\right)}\left(\left(F+\frac{\eta b}{2}\right)\beta^{\prime }-\left(F^{\prime }+\frac{b}{2}\right)\beta \right)=0.\;$$

where $Pr=\frac{\mu_{f}{\left(c_{p}\right)}_{f}}{k_{f}}$ and $M=\frac{\sigma_{nf}B_{0}^{2}x}{\rho_{f}\hat{U}}$. Moreover, f1 shows that the permeable velocity parameter and dimensionless conditions at the boundaries and away from them are in the following form:

(11) $$\begin{array}{c} \mathrm{The}\;\mathrm{conditions}\;\mathrm{for}\;\mathrm{velocity}\;\mathrm{and}\;\mathrm{temperature}\;\mathrm{at}\;\mathrm{the}\;\mathrm{surface},\;i.\;e.\;,\;\eta =0\\ F\left(\eta \right)=-f1\\ F^{\prime }\left(\eta \right)=0\\ \beta \left(\eta \right)=1\\ \mathrm{The}\;\mathrm{conditions}\;\mathrm{for}\;\mathrm{velocity}\;\mathrm{and}\;\mathrm{temperature}\;\mathrm{at}\;\eta \to \infty \\ F^{\prime }\left(\eta \right)\to 1\\ \beta \left(\eta \right)\to 0 \end{array}\},$$

The coefficient of skin friction and local Nusselt number for both types of nanofluids are as below:

(12) $$\begin{array}{c} C_{f}Re_{x}^{\frac{1}{2}}=\left(123\phi^{2}+7.3\phi +1\right)F^{\prime \prime }\left(0\right),\;\mathrm{For}\;\gamma Al_{2}O_{3}-H_{2}O\;\mathrm{Nanofluid}\\ C_{f}Re_{x}^{\frac{1}{2}}=\left(306\phi^{2}-0.19\phi +1\right)F^{\prime \prime }\left(0\right),\;\mathrm{For}\;\gamma Al_{2}O_{3}-C_{2}H_{6}O_{2}\;\mathrm{Nanofluid}\; \end{array}\},\;$$

(13) $$\begin{array}{c} \mathrm{For}\;\gamma Al_{2}O_{3}-H_{2}O\;\mathrm{Nanofluid}\;\\ N_{u}Re_{x}^{\frac{-1}{2}}=-\left(4.97\phi^{2}+2.72\phi +1\right)\beta^{\prime }\left(0\right)\\ \mathrm{For}\;\gamma Al_{2}O_{3}-C_{2}H_{6}O_{2}\;\mathrm{Nanofluid}\\ N_{u}Re_{x}^{\frac{-1}{2}}=-\left(28.905\phi^{2}+2.8273\phi +1\right)\beta \prime \left(0\right)\;\; \end{array}\},$$

## 3. Mathematical Analysis

The colloidal models γAl_2_O_3_-H_2_O and γAl_2_O_3_-C_2_H_6_O_2_ are highly nonlinear over an infinite region. However, for such flow models, closed solutions are not reliable. In such situations, it is better to tackle the models numerically. Therefore, for the purpose of solving the models under consideration (γAl_2_O_3_-H_2_O and γAl_2_O_3_-C_2_H_6_O_2_), a reliable numerical algorithm referred to as Runge-Kutta (RK) with the addition of the shooting technique ([14,15]) is adopted. For this, the fourth-order RK technique is selected. This technique is used for the system of first-order ordinary differential equations (ODEs), and the corresponding conditions at the boundaries need to be reduced into the initial conditions. Therefore, for the reduction of γAl_2_O_3_-H_2_O and γAl_2_O_3_ colloidal models into a system of first-order ODEs, the following transformations of the velocity and temperature equations are carried out:

(14) $$\begin{array}{l} {\overbrace{n}}_{1}=F,{\overbrace{n}}_{2}=F^{\prime },{\overbrace{n}}_{3}=F\prime \prime ,\;{\overbrace{n}}_{4}=\beta ,{\overbrace{n}}_{5}=\beta^{\prime }\\ {\overbrace{n}}_{1}^{\prime }=F\prime ,{\overset{\prime }{\overbrace{n}}}_{2}=F^{\prime }\prime ,{\overbrace{n}}_{3}^{\prime }=F\prime \prime \prime ,\;{\overbrace{n}}_{4}^{\prime }=\beta \prime ,{\overbrace{n}}_{5}^{\prime }=\beta^{\prime \prime }\\ {\overbrace{n}}_{1}^{\prime }={\overbrace{n}}_{2},{\overset{\prime }{\overbrace{n}}}_{2}={\overbrace{n}}_{3},{\overbrace{n}}_{3}^{\prime }=F\prime \prime \prime ,\;{\overbrace{n}}_{4}^{\prime }={\overbrace{n}}_{5},{\overbrace{n}}_{5}^{\prime }=\beta^{\prime \prime } \end{array}$$

### 3.1. γAl_2_O_3_-H_2_O Model

Firstly, reduce the γAl_2_O_3_-C_2_H_6_O_2_ nanofluid model in the following form:

(15) $$F^{\prime \prime \prime }=-\frac{1}{\varrho^{*}}\left(\left(F+\frac{\eta b}{2}\right)F^{\prime \prime }-F^{\prime 2}+b\left(F^{\prime }-1\right)+\frac{M}{\left(1-\phi +\frac{\phi {\hat{\rho }}_{s}}{{\hat{\rho }}_{f}}\right)}\left(1-F^{\prime }\right)+1\right),\;$$

(16) $$\beta^{\prime \prime }=-\frac{\mathrm{Pr}\left(\left(1-\phi +\frac{\phi {\hat{\rho }}_{s}}{{\hat{\rho }}_{f}}\right)\left(82.1\phi^{2}+3.9\phi +1\right)\right)}{\left(123\phi^{2}+7.3\phi +1\right)}\left(\left(F+\frac{\eta b}{2}\right)\beta^{\prime }-\left(F^{\prime }+\frac{b}{2}\right)\beta \right),\;$$

By means of transformations defined in Equation (14), the system in Equations (15) and (16) is reduced into the following system:

(17) $$\left[\begin{array}{c} {\overbrace{n}}_{1}^{\prime }\\ {\overbrace{n}}_{2}^{\prime }\\ {\overbrace{n}}_{3}^{\prime }\\ {\overbrace{n}}_{4}^{\prime }\\ {\overbrace{n}}_{5}^{\prime } \end{array}\right]=\left[\begin{array}{c} {\overbrace{n}}_{2}\\ {\overbrace{n}}_{3}\\ -\frac{1}{\varrho^{*}}\left[\left({\overbrace{n}}_{1}+\frac{\eta b}{2}\right){\overbrace{n}}_{3}-{\overbrace{n}}_{2}^{2}+b\left({\overbrace{n}}_{2}-1\right)+\frac{M}{1-\phi +\frac{\phi {\hat{\rho }}_{s}}{{\hat{\rho }}_{f}}}\left(1-{\overbrace{n}}_{2}\right)+1\right]\\ {\overbrace{n}}_{5}\\ -\frac{\mathrm{Pr}\left(\left(1-\phi +\frac{\phi {\hat{\rho }}_{s}}{{\hat{\rho }}_{f}}\right)\left(82.1\phi^{2}+3.9\phi +1\right)\right)}{\left(123\phi^{2}+7.3\phi +1\right)}\left[Pr\left(\left({\overbrace{n}}_{1}+\frac{\eta b}{2}\right){\overbrace{n}}_{5}-\left({\overbrace{n}}_{2}+\frac{b}{2}\right){\overbrace{n}}_{4}\right)\right] \end{array}\right],$$

where $\varrho^{*}=\frac{\left(123\phi^{2}+7.3\phi +1\right)}{\left(1-\phi +\frac{\phi {\hat{\rho }}_{s}}{{\hat{\rho }}_{f}}\right)}$.

The initial conditions can be reduced as:

(18) $$\left[\begin{array}{c} {\overbrace{n}}_{1}\\ {\overbrace{n}}_{2}\\ {\overbrace{n}}_{3}\\ {\overbrace{n}}_{4}\\ {\overbrace{n}}_{5} \end{array}\right]=\left[\begin{array}{c} -f1\\ 0\\ {\overset{˘}{m}}_{1}\\ -\frac{1}{4.97\phi^{2}+2.72\phi +1}\\ {\overset{˘}{m}}_{2} \end{array}\right],$$

where ${\overset{˘}{m}}_{1}$ and ${\overset{˘}{m}}_{2}$ are unknown and are found repeatedly unless the desired accuracy of $10^{-6}$ is attained.

### 3.2. γAl_2_O_3_-C_2_H_6_O_2_ Model

The nanofluid model for ${\gamma \mathrm{Al}}_{2}O_{3}\text{-}C_{2}H_{6}O_{2}$ can be transformed in the following manner:

(19) $$F^{\prime \prime \prime }=-\frac{1}{\varrho_{1}^{*}}\left[\left(F+\frac{\eta b}{2}\right)F^{\prime \prime }-F^{\prime 2}+b\left(F^{\prime }-1\right)+\frac{M}{\left(1-\phi +\frac{\phi {\hat{\rho }}_{s}}{{\hat{\rho }}_{f}}\right)}\left(1-F^{\prime }\right)+1\right],\;$$

(20) $$\beta^{\prime \prime }=-\frac{\mathrm{Pr}\left(\left(1-\phi +\frac{\phi {\hat{\rho }}_{s}}{{\hat{\rho }}_{f}}\right)\left(254.3\phi^{2}-3.0\phi +1\right)\right)}{\left(306\phi^{2}-0.19\phi +1\right)}\left(\left(F+\frac{\eta b}{2}\right)\beta^{\prime }-\left(F^{\prime }+\frac{b}{2}\right)\beta \right),\;$$

By using transformations, the following system is obtained:

(21) $$\left[\begin{array}{c} {\overbrace{n}}_{1}^{\prime }\\ {\overbrace{n}}_{2}^{\prime }\\ {\overbrace{n}}_{3}^{\prime }\\ {\overbrace{n}}_{4}^{\prime }\\ {\overbrace{n}}_{5}^{\prime } \end{array}\right]=\left[\begin{array}{c} {\overbrace{n}}_{2}\\ {\overbrace{n}}_{3}\\ -\frac{1}{\varrho_{2}^{*}}\left[\left({\overbrace{n}}_{1}+\frac{\eta b}{2}\right){\overbrace{n}}_{3}-{\overbrace{n}}_{2}^{2}+b\left({\overbrace{n}}_{2}-1\right)+\frac{M}{1-\phi +\frac{\phi {\hat{\rho }}_{s}}{{\hat{\rho }}_{f}}}\left(1-{\overbrace{n}}_{2}\right)+1\right]\\ {\overbrace{n}}_{5}\\ -\frac{\left(\left(1-\phi +\frac{\phi {\hat{\rho }}_{s}}{{\hat{\rho }}_{f}}\right)\left(254.3\phi^{2}-3.0\phi +1\right)\right)}{\left(306\phi^{2}-0.19\phi +1\right)}\left[Pr\left(\left({\overbrace{n}}_{1}+\frac{\eta b}{2}\right){\overbrace{n}}_{5}-\left({\overbrace{n}}_{2}+\frac{b}{2}\right){\overbrace{n}}_{4}\right)\right] \end{array}\right],$$

where $\varrho_{2}^{*}=\frac{\left(306\phi^{2}-0.19\phi +1\right)}{\left(1-\phi +\frac{\phi {\hat{\rho }}_{s}}{{\hat{\rho }}_{f}}\right)}$.

and

(22) $$\left[\begin{array}{c} {\overbrace{n}}_{1}\\ {\overbrace{n}}_{2}\\ {\overbrace{n}}_{3}\\ {\overbrace{n}}_{4}\\ {\overbrace{n}}_{5} \end{array}\right]=\left[\begin{array}{c} -f1\\ 0\\ {\overset{˘}{m}}_{1}\\ -\frac{1}{254.3\phi^{2}-3\phi +1}\\ {\overset{˘}{m}}_{2} \end{array}\right],$$

## 4. Physical Interpretation of the Results

### 4.1. Velocity

#### 4.1.1. Effects of b and f_1_ on F’(η)

**Figure 2:**
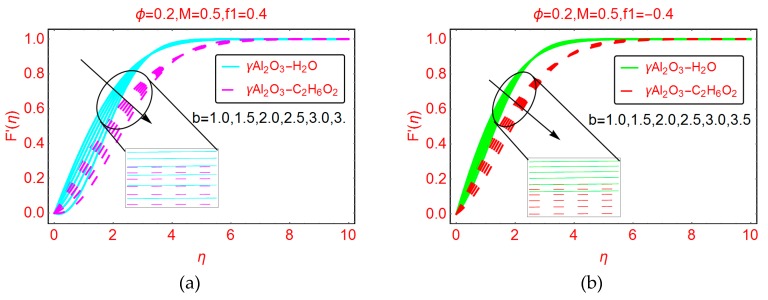
Effects of b on the velocity profile F′(η) for (**a**) f1=0.4 and (**b**) f1=−0.4.

Figure 2a,2b demonstrate the behavior of the velocity F’(η) for both γAl_2_O_3_-H_2_O and γAl_2_O_3_-C_2_H_6_O_2_ nanofluids for suction and injection, respectively. From Figure 2a, it is clear that the velocity F’(η) declines abruptly in the suction case. The drop in nanofluid velocity is very rapid near the plates. The physical reason behind the abrupt changes in the velocity F’(η) in the vicinity of the plates is that due to suction, more nanofluid particles attach to the surface. Therefore, the velocity decreases quickly near the plates. The effective dynamic viscosity of γAl_2_O_3_-C_2_H_6_O_2_ nanofluid is greater than that of γAl_2_O_3_-H_2_O nanofluid. Therefore, the nanofluid γAl_2_O_3_-C_2_H_6_O_2_ becomes denser. Due to the high density and suction of the fluid, there are more particles of γAl_2_O_3_-C_2_H_6_O_2_ nanofluid adjacent to the wall surface, and consequently, the velocity of γAl_2_O_3_-C_2_H_6_O_2_ decreases abruptly in comparison with the γAl_2_O_3_-H_2_O nanofluid. Away from the plates surface, the velocity F’(η) vanishes asymptotically for both nanofluids. Moreover, the momentum boundary layer region of γAl_2_O_3_-H_2_O increases. 

Figure 2b depicts the behavior of the velocity F’(η) when the nanofluids are injected between the plates. It can be observed that the decrement in F’(η) is quite slow when injecting the fluid. Physically, this means that for injecting fluid, the particles detached from the wall surface, and as a result the velocity F’(η) drops. This drop in velocity is slow in comparison with the suction case. Due to the lower density of γAl_2_O_3_-H_2_O, the velocity declines slowly and the boundary layer portion increases. 

**Figure 3:**
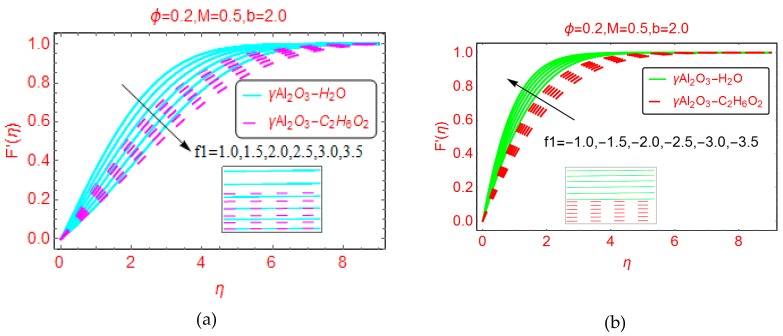
Effects of permeable parameter on the velocity F′(η) for (**a**) positive f1 and (**b**) negative f1.

The effects of suction and injection of the nanofluids between the plates are plotted in Figure 3a,b, respectively. For higher suction of the fluid, the velocity abruptly decreases. This is because, for suction of the nanofluid, more fluid layers are attached to wall surface, and due to the enhanced force of friction between the wall surface and the fluid layer, the velocity F’(η) drops prominently. Furthermore, it is noted that the boundary layer region decreases. Figure 3b shows the velocity behavior for injection of fluid. When injecting fluid, the force of friction between the fluid layer and the surface walls is reduced, and consequently the velocity F’(η) increases. The momentum boundary layer portion increases for injection of the fluid and asymptotic behavior is observed beyond η > 4.0.

#### 4.1.2. Effects of ϕ and M on F’(η)

**Figure 4:**
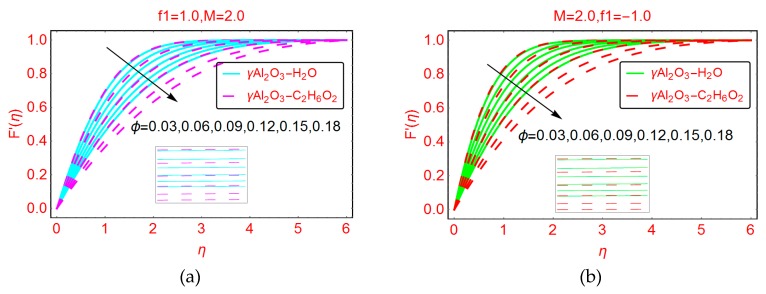
Effects of ϕ on the velocity profile F′(η) for (**a**) f1=1.0 and (**b**) f1=−1.0.

Figure 4 presents the alterations in the dimensionless velocity profile F’(η) for increasing volume fraction factor ϕ. It is worth mentioning that for the nanofluid models under consideration, the range of volume fraction is 0 < ϕ ≤ 0.2. It can be observed that the velocity F’(η) drops abruptly for higher fraction factor ϕ. Due to the high volume fraction, the density of the nanofluid under consideration increases, and the velocity starts decreasing. The physical justification of this behavior is that the density of the nanofluid is enhanced by increasing the values of volume fraction ϕ. Therefore, intermolecular forces between the nanofluid particles become stronger, and consequently the velocity drops. The boundary layer decreases for suction and increases for injection of the nanofluid. For the injecting case, the decrement in the velocity F’(η) is quite rapid. 

**Figure 5:**
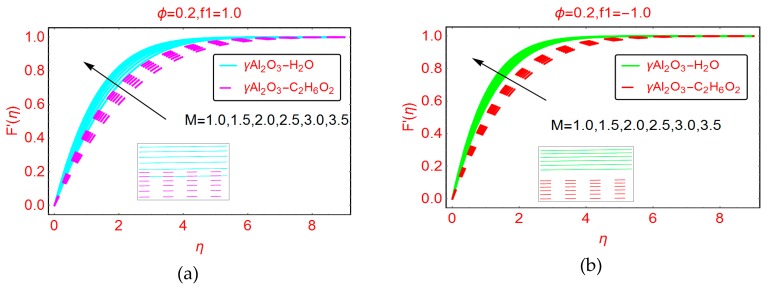
Effects of M on the velocity profile F′(η) for (**a**) f1=1.0 and (**b**) f1=−1.0.

The fascinating behavior of the magnetic parameter M on the nondimensional velocity profile F’(η) is pictured in Figure 5a,b for suction and injection, respectively. It is noted that by increasing the strength of magnetic field, the velocity F’(η) is enhanced. Due to the low density of γAl_2_O_3_-H_2_O nanofluid, the velocity increases rapidly, while slow increases in the velocity F’(η) are determined for γAl_2_O_3_-C_2_H_6_O_2_ nanofluid. Near the wall surface these variations are almost inconsequential, and prominent behavior is observed in the region 2 ≤ η ≤ 4. On the other side, injection of the fluid and varying M favors the velocity F’(η), but this increment in velocity is quite slow compared to that of the suction case.

### 4.2. Temperature

#### 4.2.1. Effects of f_1_ and b on β(η)

**Figure 6:**
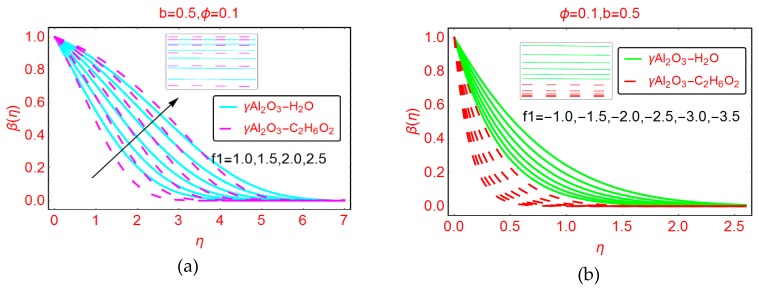
Effects of f1 on the temperature profile β(η) for (**a**) f1 positive and (**b**) f1 negative.

The effects of suction and injection on the nondimensional temperature profile β(η) for γAl_2_O_3_-H_2_O and γAl_2_O_3_-C_2_H_6_O nanofluid pictured in Figure 6a,b, respectively. The nanofluid temperature β(η) abruptly increases for suction of the fluid. For the suction phenomena, the nanofluid layer is closest to the wall surface, and due to the force of friction between the surface and the nanofluid layer, the temperature β(η) is enhanced. The thermal boundary layer region for γAl_2_O_3_-H_2_O nanofluid increases. The increasing behavior of the temperature near the surface is almost negligible for both types of nanofluids under consideration. In the region 1 < η < 3, the temperature increases abruptly. The temperature vanishes asymptotically at large from the plates. 

Figure 6b portrays the temperature behavior β(η) for the injections of the fluid. For the injection case, the temperature β(η) decreases very quickly. For γAl_2_O_3_-H_2_O nanofluid, a rapid decreasing behavior of the nanofluid temperature was determined. Because the thermal conductivity and effective density of γAl_2_O_3_-C_2_H_6_O_2_ is higher than that of γAl_2_O_3_-H_2_O, the fluid motion is decreased due to the high density of γAl_2_O_3_-C_2_H_6_O_2_ nanofluid, therefore causing the temperature to drop abruptly. Furthermore, it can be noticed that the thermal boundary layer region was increased for γAl_2_O_3_-C_2_H_6_O_2_ and asymptotically vanished beyond η > 1. For γAl_2_O_3_-H_2_O nanofluid the thermal boundary layer region decreased, and asymptotic behavior of β(η) was observed for η ≥ 2.5. 

**Figure 7:**
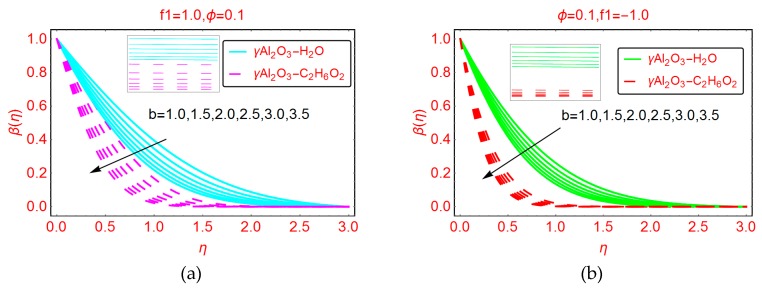
Effects of b on the temperature profile β(η) for (**a**) f1=1.0 (**b**) f1=−1.0.

The variations in dimensionless temperature β(η) by varying b are depicted in Figure 7a,b for suction and blowing cases, respectively. It is noted that the temperature drops rapidly for suction of the fluid. Due to the high density of γAl_2_O_3_-C_2_H_6_O_2_ nanofluid, the temperature decreases abruptly and the low density of γAl_2_O_3_-H_2_O nanofluid leads to a comparatively slow decrease in the temperature. On the other side, slow decreasing behavior of β(η) is observed for injecting of the fluid. Furthermore, it can be observed that the thermal boundary layer increases when injecting fluid and decreases for the suction case.

#### 4.2.2. Effects of ϕ on β(η)

**Figure 8:**
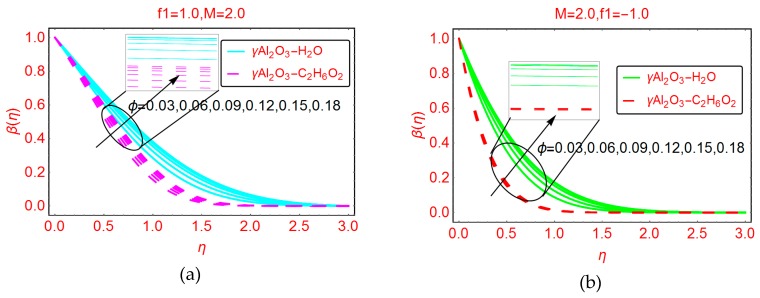
Effects of ϕ on the temperature profile β(η) (**a**) f1=1.0 (**b**) f1=−1.0.

The volume fraction factor of the nanoparticles is the key parameter for the enhancement of temperature in the nanofluid. These effects are plotted in Figure 8a,b for the suction and blowing cases, respectively. The volume fraction ϕ enhances the temperature for both suction and blowing of the under consideration nanofluids. The increase in temperature β(η) is clear and prominent in the suction case. For blowing of the nanofluid, the thermal boundary layer increases for γAl_2_O_3_-C_2_H_6_O_2_ nanofluid and the temperature increases very slowly.

### 4.3. Streamlines and Isotherms Profile

**Figure 9:**
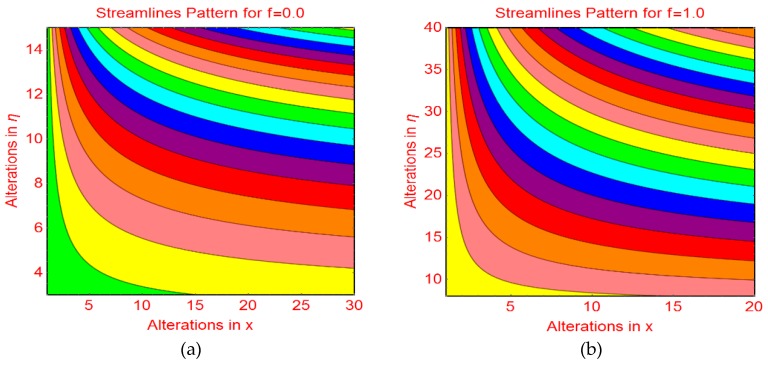
Streamlines profile for (**a**) f1=0.0 and (**b**) f1=1.0.

**Figure 10:**
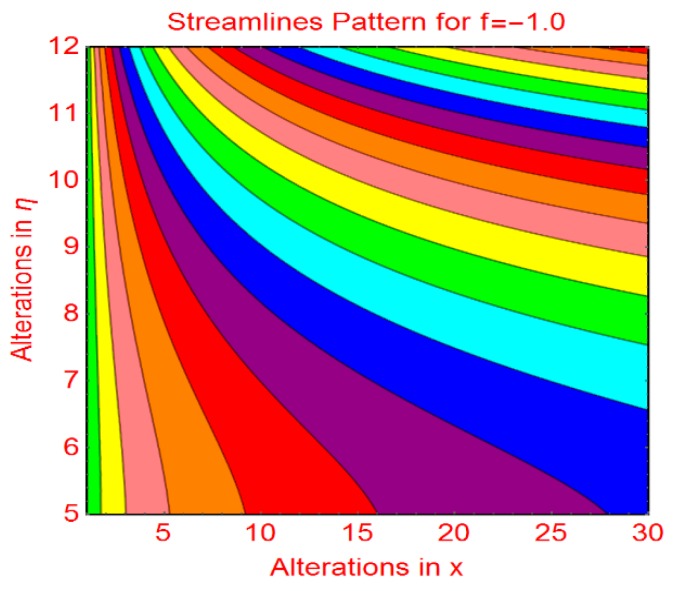
Streamlines profile for f1=−1.0.

**Figure 11:**
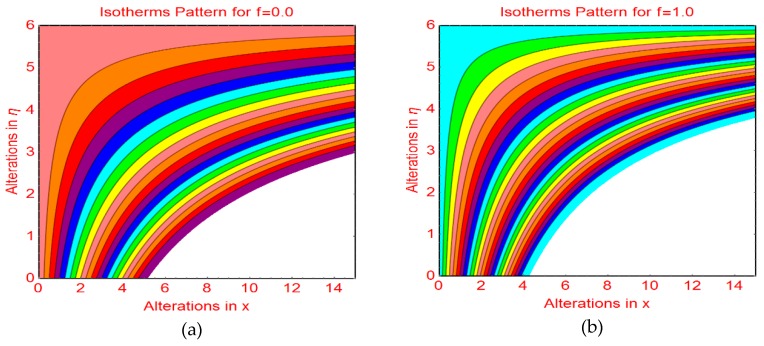
Isotherms profile for (**a**) f1=0.0 (**b**) f1=1.0.

**Figure 12:**
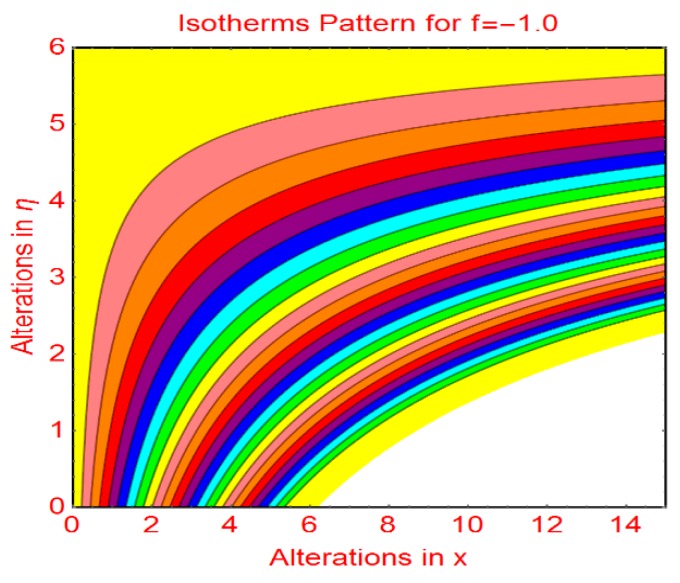
Isotherms profile for f1=−1.0.

This subsection addresses the streamline and isotherm profiles for suction and blowing cases. The profile of streamlines is more curved for permeable parameter f_1_ = 0 and f_1_ = 1.0. These are plotted in Figure 9a,b, respectively. The flow pattern becomes flat far from the wall surface. For injecting fluid, fascinating streamlines profile is observed in Figure 10. Figure 11 and Figure 12 highlight the fascinating isotherm profile for different permeable parameters f_1_.

### 4.4. Thermophysical Properties

**Figure 13:**
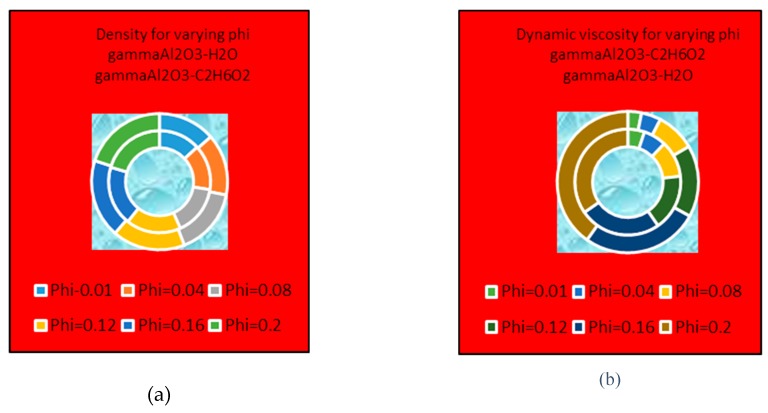
Effects of ϕ on (**a**) dynamic viscosity (**b**) thermal conductivity.

**Figure 14:**
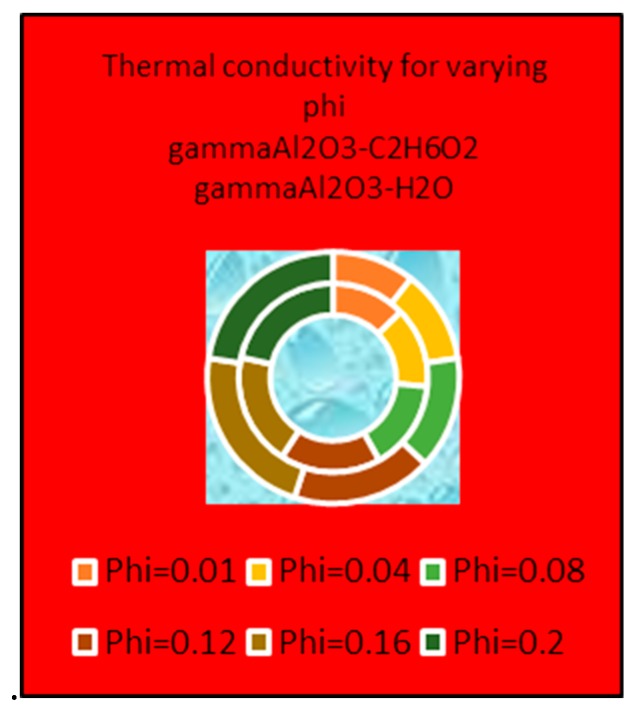
Effects of ϕ on effective thermal conductivity.

Figure 13 and Figure 14 present the effects of volume fraction factor ϕ on the effective density, dynamic viscosity and thermal conductivity of γAl_2_O_3_-H_2_O and γAl_2_O_3_-C_2_H_6_O_2_. It is shown that the effective density of γAl_2_O_3_ H_2_O is larger than that of γAl_2_O_3_ C_2_H_6_O_2_. Meanwhile, dominating behavior of dynamic viscosity is noted for γAl_2_O_3_-C_2_H_6_O_2_ nanofluid. These effects are pictured in Figure 13a,b, respectively. Furthermore, it can be observed that thermal conductivity of the nanofluid improves with increasing ϕ. In Figure 14, the thermal conductivity of both types of nanofluids is enhanced but, for γAl_2_O_3_-C_2_H_6_O_2_, it shows dominating behavior.

### 4.5. Engineering Quantities

**Figure 15:**
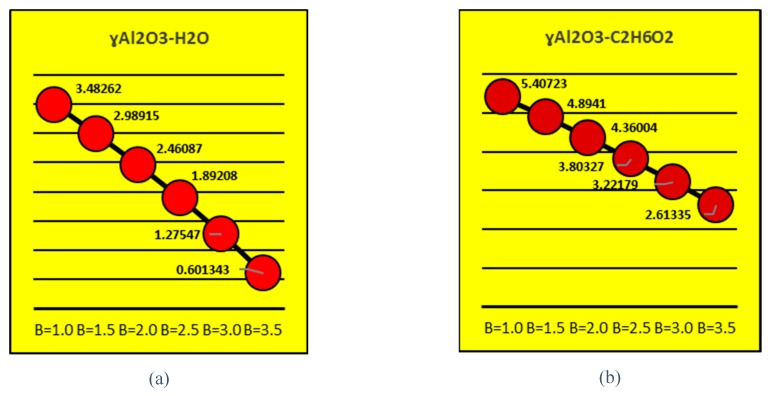
Effects of b on the coefficient of skin friction (**a**) ${\gamma \mathrm{Al}}_{2}O_{3}\text{-}H_{2}O$ and (**b**) ${\gamma \mathrm{Al}}_{2}O_{3}\text{-}C_{2}H_{6}O_{2}$.

**Figure 16:**
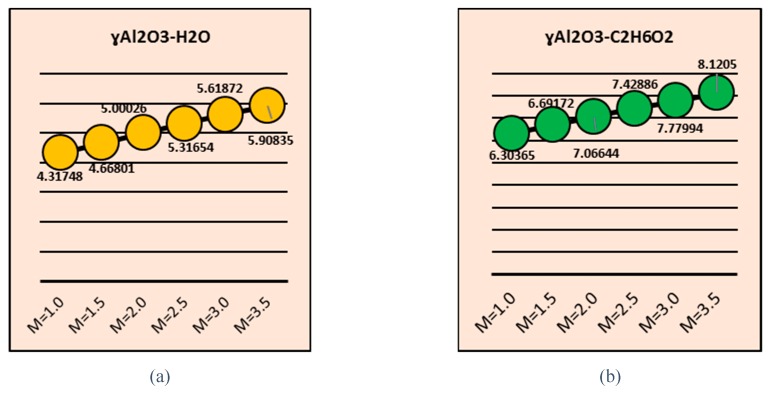
Effects of M on the coefficient of skin friction (**a**) ${\gamma \mathrm{Al}}_{2}O_{3}\text{-}H_{2}O$ and (**b**) ${\gamma \mathrm{Al}}_{2}O_{3}\text{-}C_{2}H_{6}O_{2}$.

The behavior of the coefficient of skin friction for different values of b and M is demonstrated in Figure 15 and Figure 16, respectively. The results are plotted for both nanofluids. The wall shear stresses increase abruptly for γAl_2_O_3_-H_2_O nanofluid, and quite slow behavior of shear stresses is determined for ethylene glycol-based nanofluid suspended by γ-nanoparticles. These results are pictured in Figure 15. Through variation, almost identical shear stress behaviors were observed for both water and ethylene glycol nanofluids composed of γ-nanoparticles. These effects can be seen from Figure 15. Figure 16 decorated the wall shear stress for multiple values of magnetic number M. It is examined that for stronger magnetic field, wall shear stresses increases. 

### 4.6. Reliability of the Study

Table 1 presents the reliability of the study on two different types of nanofluids between parallel plates. It can be seen that under certain parameter conditions, our results are acceptable based on comparison with the results of [5], which proves the applicability of the adopted numerical study and the reliability of the presented analysis.

## 5. Conclusions

The flow of water and ethylene glycol (EG) composed of γ-nanoparticles between two infinite parallel plates suspended by micro-cantilever sensors was considered. On the basis of the effective Prandtl model, the governing model was transformed into two different nanofluid flow models. The models were treated numerically and fascinating results were found for the velocity F’(η), temperature β(η), streamlines and isotherms pattern. It was determined that:

- For higher values of the parameter b, the velocity profile F’(η) drops, and there is an abrupt decrement of suction of the nanofluid between the plates.
- In the case of suction, the velocity of γAl_2_O_3_-H_2_O and γAl_2_O_3_-C_2_H_6_O_2_ decreased rapidly, while a slow decrement was observed when injecting fluid.
- The momentum boundary layer region increased with blowing of the fluid and decreased in the suction case.
- The volume fraction factor ϕ opposes the velocity F’(η) and decreased very rapidly for both suction and blowing of the fluid.
- The temperature field β(η) was enhanced with higher suction parameters and reduced with blowing of the fluid.
- The volume fraction ϕ favors the temperature of γAl_2_O_3_-H_2_O and γAl_2_O_3_-C_2_H_6_O_2_ nanofluids.
- The thermophysical characteristics effective density, dynamic viscosity and thermal conductivity increased with increasing volume fraction ϕ.
- The coefficient of skin friction of γAl_2_O_3_-H_2_O nanofluid increased abruptly in comparison with γAl_2_O_3_-C_2_H_6_O_2_ at higher values of b.
- Our results were acceptable under different conditions of flow parameters on the basis of comparison with already-existing literature.

## Figures and Tables

**Table 1:** Reliability of the presented analysis (numerical values for skin friction coefficient).

$f_{1}=-0.5,\;M=0.5,\;b=1.0$		
ϕ	[5]	Present Results
0.0	1.481134	1.48113
$f_{1}=0.5,\;M=0.5,\;b=0.0$		
ϕ	[5]	Present Result
0.0	1.162236	1.16224
